# Pseudotumor cerebri syndrome causing a terson like syndrome

**DOI:** 10.1016/j.ajoc.2020.100993

**Published:** 2020-11-24

**Authors:** Joseph Raevis, Valerie I. Elmalem

**Affiliations:** a State University of New York Downstate Medical Center, 450 Clarkson Ave, Brooklyn, NY, 11203, United States; b New York Eye and Ear Infirmary of Mount Sinai, 310 E 14th Street, New York, NY, 10003, United States

**Keywords:** Pseudotumor cerebri syndrome, Idiopathic intracranial hypertension, Terson syndrome, Vitreous hemorrhage, Papilledema, Vitreous Hemorrhage

## Abstract

**Purpose:**

Terson syndrome presents with retinal and vitreous hemorrhages in patients with a subarachnoid hemorrhage or after acutely elevated intracranial pressure. The source of this hemorrhage has been debated and may originate either from direct extension of intracranial hemorrhage or more likely from the peripapillary retinal vessels.

**Observations:**

A 39-year-old woman presenting with nausea, vomiting, floaters and papilledema with normal neuroimaging was diagnosed ultimately with pseudotumor cerebri syndrome. She had a right vitreous hemorrhage and bilateral subretinal and intraretinal hemorrhages which were consistent with Terson like syndrome. Her symptoms resolved with acetazolamide over one month and the retinal and vitreous hemorrhages significantly improved over two months.

**Conclusions and importance:**

This case with initial presentation of pseudotumor cerebri syndrome causing a Terson like syndrome may help elucidate the mechanism behind the etiology of these hemorrhages from leaking peripapillary vessels.

## 1 Introduction

Pseudotumor cerebri syndrome (PTCS) can be primary (idiopathic intracranial hypertension) or due to secondary causes. A diagnosis of PTCS is probable if a patient has papilledema, normal cerebral spinal fluid (CSF) composition, normal neuroimaging (except for evidence of elevated intracranial pressure) and an intact neurologic examination except for cranial nerve abnormalities. The diagnosis is considered definitive if the prior criteria are met and a lumbar puncture (LP) reveals an open pressure of ≥250 mm CSF in adults or ≥ 280 mm CSF in children.^1^ We present a rare case of a patient with initial presentation of definitive primary PTCS causing a Terson like syndrome.

### 1.1 Case report

A 39-year-old woman with rheumatoid arthritis, sleep apnea and a body mass index of 55 presented with nausea, vomiting and floaters in the right eye for two days. Two weeks prior, she developed positional headaches, blurry vision and transient visual obscurations. On presentation, her best corrected visual acuity was 20/25 in each eye, there was no relative afferent pupillary defect and the examination of external structures and anterior segment was unremarkable.

**Fig. 1:**
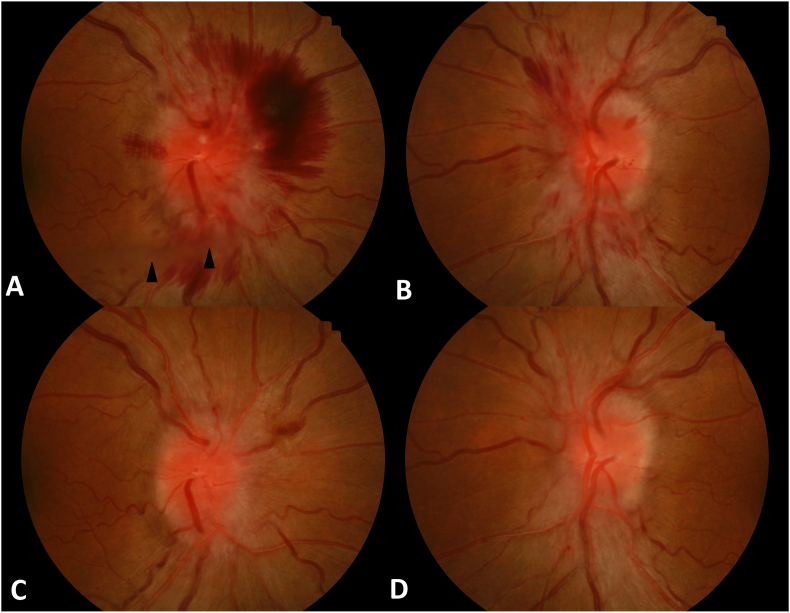
Color fundus photographs showing bilateral dilated tortuous veins and flame shaped hemorrhages. (A) There was right Frisen grade 4 papilledema with mild obscuration of the major vessels on the disc and flame shaped hemorrhages. A small vitreous hemorrhage can be seen inferiorly (arrowheads) and a superior nasal peripapillary subretinal and intraretinal hemorrhage is also present. (B) Grade 3 papilledema and intraretinal flame shaped hemorrhages are seen in the left eye. At two-month follow up, there was resolution of the vitreous hemorrhage, flame-shaped hemorrhages, and marked improvement in papilledema of the right (C) and left (D) eyes.

**Fig. 2:**
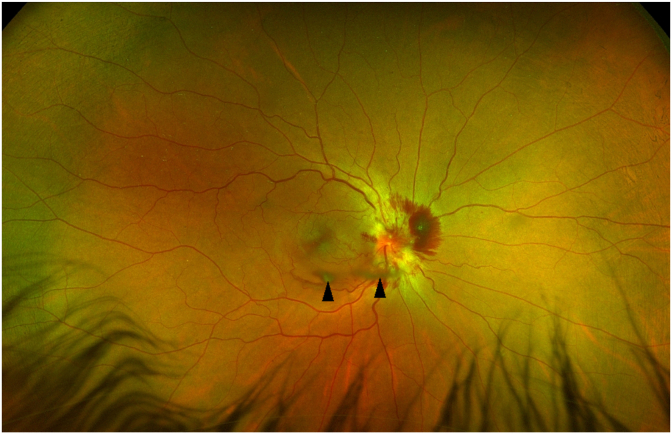
Wide angle color fundus photograph of the right eye on presentation showing the extent of the vitreous hemorrhage and normal peripheral retina.

Funduscopic examination revealed Frisen grade 4 papilledema in the right eye with vitreous, subretinal and intraretinal hemorrhages, and grade 3 in the left eye with flame-shaped hemorrhages (Fig. 1A, Fig. 2).

Head CT with contrast appeared normal, an MRI was not possible due to body habitus and weight restrictions. Lumbar puncture under fluoroscopic guidance revealed opening pressure of 310 mm CSF with normal composition. Humphrey visual field 24-2 SITA Fast was reliable with an enlarged blind spot and a few central changes in the right eye (mean deviation −4.00 dB) and was essentially full with mild central depression in the left eye (mean deviation −3.55 dB).

She was started on acetazolamide 500 mg twice daily, which was titrated up to 1500 mg twice daily with gradual resolution of symptoms over a month. At two month follow up, the vitreous hemorrhage had resolved and there was marked improvement in the papilledema bilaterally (Fig. 1 C, D).

## 2 Discussion

Terson syndrome is known to present with subretinal, sub-internal limiting membrane, subhyaloid and vitreous hemorrhages and is classically associated with subarachnoid hemorrhages or trauma. Cases of Terson syndrome, or a Terson-like syndrome as some authors like to call it, have been documented to occur during acute elevations in intracranial pressure (ICP) such as following epidural saline injection,^2^ neurosurgical third ventriculostomy,^3^ in pediatric patients,^4^ in those with papilledema with optociliary shunt vessels^5^ and PTCS.^6^ Cases have also been reported in patients with viral meningoencephalitis^7^ and traumatic head injury.^4^

Our patient with PTCS is significant because she presented acutely with a vitreous, subretinal and intraretinal hemorrhages, not associated with optociliary shunt vessels, trauma or surgical procedure. A study by Keane^6^ found two patients with PTCS out of 402 consecutive patients with papilledema and vitreous hemorrhages. One patient had only a vitreous hemorrhage while the second case had retinal hemorrhages and vitreous hemorrhage similar to this case. Here we show a patient similar to Keane's latter patient, but we show resolution of the hemorrhages with optic disc and wide field Optos images. With treatment of the elevated ICP, vitreous hemorrhage and retinal hemorrhages will resolve with time.^6^

Our patient's retinal hemorrhages are unlikely due to a hyperviscosity retinopathy as those findings tend to be distributed throughout the posterior pole, rather than centered around the optic nerve. No signs of proliferative diabetic retinopathy were seen on fundoscopic examination. Peripapillary choroidal neovascularization may be a cause of retinal hemorrhages and is associated with macular degeneration, polypoidal choroidal vasculopathy, high myopia, traumatic choroidal ruptures and inflammatory causes such as birdshot retinochoroidaopathy.^8^ However, peripapapillary choroidal neovascularization is unlikely to be bilateral, especially in a young patient without inflammatory causes. Valsalva retinopathy tends to present with preretinal hemorrhages due to a sudden increase in intrathoracic or abdominal pressure. While she did not have a history of trauma it is possible vomiting could have induced or aggravated the intraocular hemorrhages.

The source of hemorrhage in Terson syndrome is debated in the literature. Subarachnoid hemorrhage within the optic nerve may enter the sub-ILM region through the perivascular space surrounding retinal vessels (Virchow-Robin spaces).^9^^,^^10^ This mechanism explains how a subarachnoid hemorrhage could enter the eye; however, would not explain how acute rises in ICP can cause intraocular hemorrhage.

An alternative mechanism, and more likely mechanism, is that an acute rise in ICP causes CSF within the optic nerve sheath to directly compress the central retinal vein. The resulting venous hypertension leads to rupture of the veins in the peripapillary region^7^^,^^11^^,^^12^ causing vitreous, subretinal and intraretinal hemorrhages.^13^ This mechanism explains the findings in our patient and is further supported by an intraoperative fluorescein angiography taken during a pars plana vitrectomy showing dye leakage from what appeared to be a ruptured peripapillary vein or hemorrhage from the underlying neurosensory retina that hydrodissected the internal limiting membrane to enter the vitreous cavity.^9^

A histopathologic study on autopsies revealed evidence that there can be multiple foci of hemorrhages throughout the dural sheath, subarachnoid space or pia mater and throughout the optic nerve itself in addition to the eye. These findings have supported the theory that the hemorrhages are from multiple sites of vessel bleeding, rather than from a direct extension of blood from the subarachnoid space. This study also found that subhyaloid bleeding is diffuse, with irregular edges, while sub internal limiting hemorrhages are dome shaped and well-demarcated.^13^

## 3 Conclusions

This is a rare case of definitive diagnosis of PTCS causing a Terson like syndrome. We presume the paucity of prior reports of PTCS with vitreous hemorrhage is due to the majority having gradual rise in ICP rather than acute elevation. This case lends further support that an elevated CSF pressure may compress the central retinal vein, causing venous hypertension and rupture of the peripapillary vessels resulting in retinal and vitreous hemorrhages. Lastly, patients with retinal hemorrhages and a mild vitreous hemorrhage in Terson like syndrome may be conservatively managed without need for vitrectomy.

## Patient consent

Consent to publish the case report was not obtained. This report does not contain any personal information that could lead to the identification of the patient.

## Acknowledgments and disclosures

No funding or grant support.

## Authorship

All authors attest that they meet the current ICMGE criteria for Authorship.

## Declaration of competing interest

The following authors have no financial disclosures (JR, VE)
